# Modelling the Use of Vaccine and *Wolbachia* on Dengue Transmission Dynamics

**DOI:** 10.3390/tropicalmed5020078

**Published:** 2020-05-13

**Authors:** Meksianis Z. Ndii

**Affiliations:** Department of Mathematics, Faculty of Sciences and Engineering, University of Nusa Cendana, Kupang 85361, Indonesia; meksianis.ndii@staf.undana.ac.id

**Keywords:** mathematical model, dengue, vaccine, *Wolbachia*

## Abstract

The use of vaccine and *Wolbachia* has been proposed as strategies against dengue. Research showed that the *Wolbachia* intervention is highly effective in areas with low to moderate transmission levels. On the other hand, the use of vaccine is strongly effective when it is implemented on seropositive individuals and areas with high transmission levels. The question that arises is could the combination of both strategies result in higher reduction in the number of dengue cases? This paper seeks to answer the aforementioned question by the use of a mathematical model. A deterministic model in the presence of vaccine and *Wolbachia* has been developed and analysed. Numerical simulations were presented and public health implications were discussed. The results showed that the performance of *Wolbachia* in reducing the number of dengue cases is better than that of vaccination if the vaccine efficacy is low, otherwise, the use of vaccine is sufficient to reduce dengue incidence and hence the combination of *Wolbachia* and vaccine is not necessary.

## 1. Introduction

Dengue is a vector-borne disease with around 390 million cases annually and mostly occurs in tropical and sub-tropical regions [1]. An increase in dengue cases has been noticed with a 30-fold increase in incidence over the past 50 years [2]. Dengue is caused by four different serotypes where individuals generally obtain lifelong immunity to the serotype they are infected with, although reinfection with the same serotype is possible [3]. The secondary infection may be worse as it can increase the risk of severe disease [4].

A number of strategies such as insecticides have been implemented, but they are unsustainable [5] and hence alternative strategies are required. The current proposed strategies are by the use of vaccine and *Wolbachia* bacterium. Around 86% of dengue reduction can be obtained by the use of *Wolbachia* bacterium in particular if it is implemented in regions with low to moderate transmission level [6,7,8,9,10,11,12]. The use of vaccine can reduce the number of dengue cases up to 80% depending on individual ages and status (seronegative or seropositive) and the transmission level in the regions [13,14]. CYD-TDV is the only dengue vaccine licensed to date [15]. Several trials have shown satisfactory safety profile of the vaccine [16,17] and balanced immune response to the vaccine [18,19]. An analysis of multiple phase-2 trials of CYD-Tetravalent Dengue Vaccine (CYD-TDV) showed the importance of dengue exposure prior to vaccination on the vaccine immunogenicity. Furthermore, research showed the distinct vaccine efficacy against dengue serotypes with no significant efficacy against serotype 2 [20,21] and a decrease in protective effects in years 3 and 4 after vaccination [22]. This may increase the risk of the use of the vaccine [13]. Therefore, the implementation of vaccination strategy should be carefully designed and considers important factors such as vaccination age, doses, and individual status (seronegative or seropositive) [13,17,21]. Although research and development of both strategies are still underway, understanding the combination of these interventions before they are publicly used is important. Understanding the complex phenomena by using a mathematical model is common. Many mathematical models have been widely formulated to understand dengue transmission dynamics and measure the effectiveness of *Wolbachia* and vaccination in reducing the number of dengue cases [6,7,8,9,11,23,24,25,26,27,28]. Ndii et al. [7,8,9] formulated a dengue mathematical model in the presence of *Wolbachia* and assessed the effectiveness of *Wolbachia* intervention in reducing dengue transmission. They found that the *Wolbachia* can reduce the number of dengue cases up to 80% particularly in regions with low to moderate transmission level. The results were similar to that found by Ferguson et al. [6]. O’ Reilly et al. used a mathematical model to assess the performance of *Wolbachia* in reducing dengue transmission in Indonesia and found 80% reduction in dengue cases [11]. Furthermore, a long-term implementation of *Wolbachia* provided a higher reduction in dengue incidence [26]. Aguiar et al. [14] investigated the effects of vaccination on dengue transmission dynamics and found that, if the vaccine is implemented on partial immune individuals, a significant reduction in disease burden can be obtained. Using a mathematical model, Ferguson et al. [13] showed the benefits and risks of using dengue vaccine with an increase risk of hospitalization if the vaccination is implemented in regions with low to moderate transmission levels, and the benefits if it is implemented in regions with a high transmission level. Due to different factors affecting the performance of these interventions, it has been suggested to combine both strategies [12]. A question that then arises is “could the combination of both strategies result in higher reduction in the number of dengue cases?” This paper seeks to answer the aforementioned question by the use of a mathematical model.

Although many mathematical models have been formulated to study dengue transmission dynamics in the presence of vaccine and *Wolbachia*, they did not take into account the combination of both strategies. It is important to compare the performance of both interventions individually and the combination of them. To date, only a few research papers have been conducted to examine the effects of the combination between vector control and vaccine. Hladish et al. [29] have recently investigated the performance of the combination of vaccine and vector control (insecticide-based method) in minimizing the dengue transmission and found that the combination of strategies outperformed single intervention. Different to Hladish et al. [29], in this paper, we investigate the performance of the vaccine and *Wolbachia* in reducing dengue transmission. *Wolbachia* has different characteristics to the insecticide-based method which may affect the disease transmission dynamics. We will show the performance of both strategies individually and the combination of them. The aim of the paper is to obtain general understanding of the effectiveness of the strategy and hence a single serotype dengue model is sufficient.

## 2. Methods and Results

### 2.1. Formulation of the Mathematical Model

A deterministic mathematical model in the presence of *Wolbachia* and vaccination is formulated, which is the extension of a dengue mathematical model in the presence of *Wolbachia* formulated by Ndii et al. [8]. This is a compartment-based model where the human and mosquito populations are divided into different compartment depending on their disease status. The human population is divided into susceptible ($S_{H}$), vaccinated ($V_{H}$), exposed ($E_{H}$), infectious ($I_{H}$), and recovered ($R_{H}$). Mosquito population is divided into aquatic (*A*), susceptible (*S*), exposed (*E*), infectious (*I*) with subscripts *N* and *W* denoting non-*Wolbachia* and *Wolbachia* mosquitoes, respectively. There is no recovered class for mosquitoes as they remain infectious for the rest of their life. As the aim of the paper is to gain general insights of the possible effectiveness of the use of vaccine and *Wolbachia*, the use of a single serotype dengue model is sufficient. An extension of this work to investigate the serotype-specific effects on the effectiveness of the use of both strategies is the subject of the future work.

The susceptible individuals are infected when they are bitten by infected non-*Wolbachia* and *Wolbacbia*-carrying mosquitoes at a rate of $\lambda_{N}$ and $\lambda_{W}$, respectively. The human population is vaccinated at a rate $v_{h}$ and the vaccinated individuals are exposed to dengue when the vaccine loss its efficacy at a rate (1−ϵ) and the individuals are bitten by infected non-*Wolbachia* and *Wolbachia*-carrying mosquitoes at a rate $\lambda_{N}$ and $\lambda_{W}$, respectively. We take into account the waning immunity which happens at a rate of $\phi_{h}$ and the random mass vaccination.

The model is governed by the following system of differential equations:

(1) $$\begin{array}{cc} \frac{dS_{H}}{dt} & =BN_{H}-pS_{H}-\lambda_{N}S_{H}-\lambda_{W}S_{H}-\mu_{H}S_{H}+\phi V_{H},\\ \frac{dV_{H}}{dt} & =pS_{h}-\left(1-\epsilon \right)\lambda_{N}V_{H}-\left(1-\epsilon \right)\lambda_{W}V_{H}-\phi V_{H}-\mu_{H}V_{H},\\ \frac{dE_{H}}{dt} & =\lambda_{N}S_{H}+\lambda_{W}S_{H}+\left(1-\epsilon \right)\lambda_{N}V_{H}+\left(1-\epsilon \right)\lambda_{W}V_{H}-\gamma_{H}E_{H}-\mu_{H}E_{H},\\ \frac{dI_{H}}{dt} & =\gamma_{H}E_{H}-\sigma I_{H}-\mu_{H}S_{H},\\ \frac{dR_{H}}{dt} & =\sigma I_{H}-\mu_{H}R_{H},\\ \frac{dA_{N}}{dt} & =\rho_{N}\frac{F_{N}^{2}}{2(F_{N}+F_{W})}\left(1-\frac{\left(A_{N}+A_{W}\right)}{K}\right)-\left(\tau_{N}+\mu_{NA}\right)A_{N},\\ \frac{dS_{N}}{dt} & =\tau_{N}\frac{A_{N}}{2}+\left(1-\alpha \right)\tau_{W}\frac{A_{W}}{2}-\left(\frac{b_{N}T_{N}I_{H}}{N_{H}}+\mu_{N}\right)S_{N},\\ \frac{dE_{N}}{dt} & =\frac{b_{N}T_{N}I_{H}}{N_{H}}S_{N}-\left(\gamma_{N}+\mu_{N}\right)E_{N},\\ \frac{dI_{N}}{dt} & =\gamma_{N}E_{N}-\mu_{N}I_{N},\\ \frac{dA_{W}}{dt} & =\rho_{W}\frac{F_{W}}{2}\left(1-\frac{\left(A_{N}+A_{W}\right)}{K}\right)-\left(\tau_{W}+\mu_{WA}\right)A_{W},\\ \frac{dS_{W}}{dt} & =\tau_{W}\alpha \frac{A_{W}}{2}-\left(\frac{b_{W}T_{N}I_{H}}{N_{H}}+\mu_{W}\right)S_{W},\\ \frac{dE_{W}}{dt} & =\frac{b_{W}T_{N}I_{H}}{N_{H}}S_{W}-\left(\gamma_{W}+\mu_{W}\right)E_{W},\\ \frac{dI_{W}}{dt} & =\gamma_{W}E_{W}-\mu_{W}I_{W}, \end{array}$$

where

(2) $$\begin{array}{cc} \lambda_{N} & =\frac{b_{N}T_{N}I_{N}}{N_{H}},\\ \lambda_{W} & =\frac{b_{W}T_{HW}I_{W}}{N_{H}}. \end{array}$$

The description of the parameters, references, and values are given in Table 1.

Using the concept of the next generation matrix, we obtain the basic reproduction number which is the average number of new infections generated by one infectious individual in the entirely susceptible population. The basic reproduction number in the absence of interventions is given by

(3) $$R_{A}=\sqrt{\frac{b_{N}^{2}T_{N}^{2}\gamma_{N}\gamma_{H}S_{N}}{\mu_{N}\left(\gamma_{N}+\mu_{N}\right)\left(\sigma +\mu_{H}\right)\left(\gamma_{H}+\mu_{H}\right)N_{H}}}.$$

### 2.2. Sensitivity Analysis

We performed a global sensitivity analysis to determine the most influential parameters of the model by using the combination of a Latin Hypercube Sampling (LHS) and Partial Rank Correlation Coefficient (PRCC) [42]. We measure against the increasing number of infected individuals, which is the solution of

(4) $$\frac{dC_{I_{H}}}{dt}=\gamma_{H}E_{H}.$$

**Figure 1:**
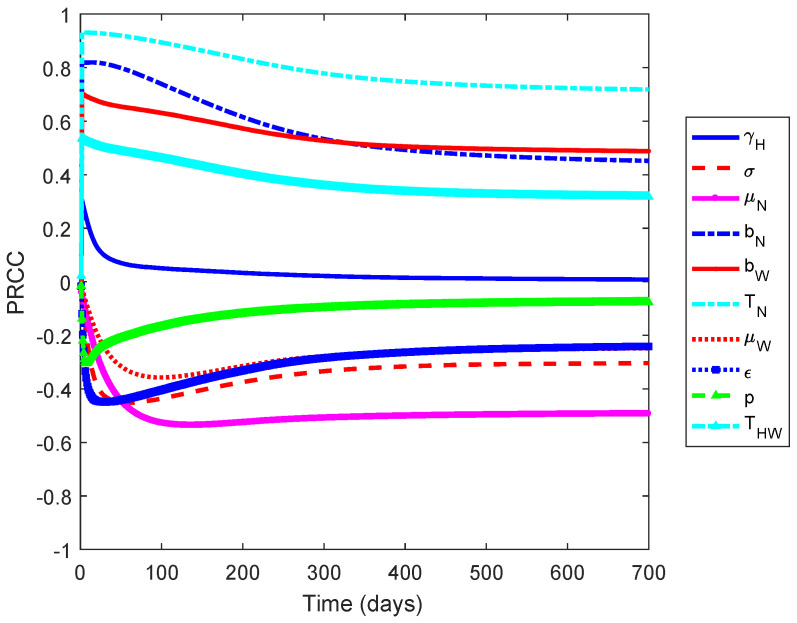
Partial Rank Correlation Coefficient (PRCC) values for the model when measured against the increasing number of infected individuals. The positive and negative values indicate the relationship of the parameter and an increase in the number of infected individuals.

Figure 1 shows that the non-*Wolbachia* and *Wolbachia*-carrying mosquitoes death rates ($\mu_{N}$ and $\mu_{W}$), vaccine efficacy (ϵ), the biting rates ($b_{N}$ and $b_{W}$), and the transmission probability ($T_{N}$) are the most influential parameters on the increased number of infected individuals. The first three parameters have negative relationships and the latter have positive relationships. This implies that an increase in mosquito death rates and the vaccine efficacy results in the reduction of the number of infected individuals. Moreover, a decrease in the biting rates and the transmission probabilty leads to the reduction in the number of dengue cases.

### 2.3. Numerical Simulations

#### 2.3.1. Dengue Reduction

Here, we present the dengue reduction with three different scenarios: Vaccination only, *Wolbachia* only, and both vaccination and *Wolbachia*. We also show the numerical solutions of the model with different vaccination rate and vaccine efficacy.

**Figure 2:**
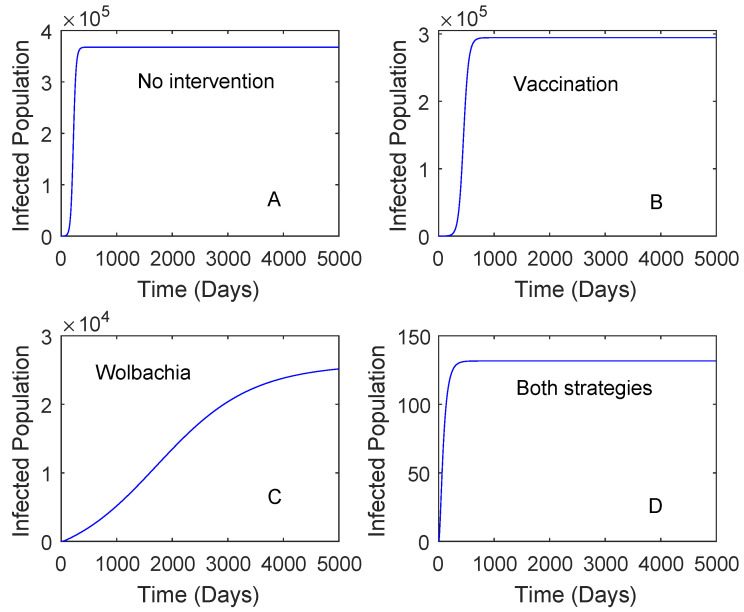
Numerical simulations of the model with no intervention, vaccine only, *Wolbachia* only, and both vaccine and *Wolbachia*. This is for the case $R_{A}=2.91$. The vaccine efficacy is 0.536 and the vaccination rate is 0.2. Plot (**A**): No intervention, Plot (**B**): Vaccination, Plot (**C**): *Wolbachia*, Plot (**D**): Both *Wolbachia* and vaccination strategies.

Figure 2 presents the numerical solutions of the model where the vaccine efficacy and the vaccination rate are 0.536 and 0.2, respectively. The vaccine efficacy of 0.536 represents the effectiveness of vaccine on seronegative individuals. The result showed that the use of *Wolbachia* only reduces a higher number of dengue cases in comparison to that of the vaccine. The use of vaccination only, *Wolbachia* only, and both strategies can reduce the number of dengue cases around 19%, 92%, and 99%, respectively. This suggests that the use of *Wolbachia* is sufficient to reduce the number of dengue cases if the vaccine efficacy is low.

**Figure 3:**
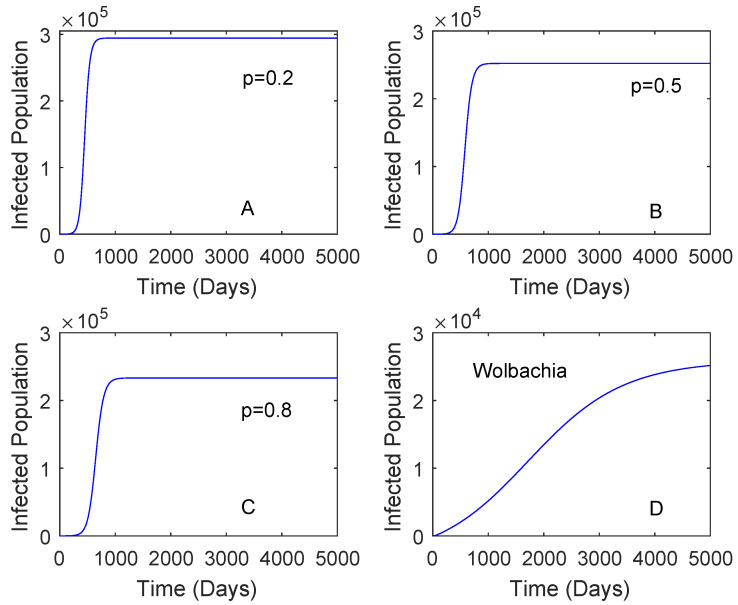
Comparing the performance of vaccination and *Wolbachia* with different vaccination rates. This is for the case $R_{A}=2.91$ and the vaccine efficacy is 0.536. The vaccination rates are 0.2 plot (**A**), 0.5 plot (**B**), 0.8 Plot (**C**). Plot (**D**) is for *Wolbachia* strategy.

**Figure 4:**
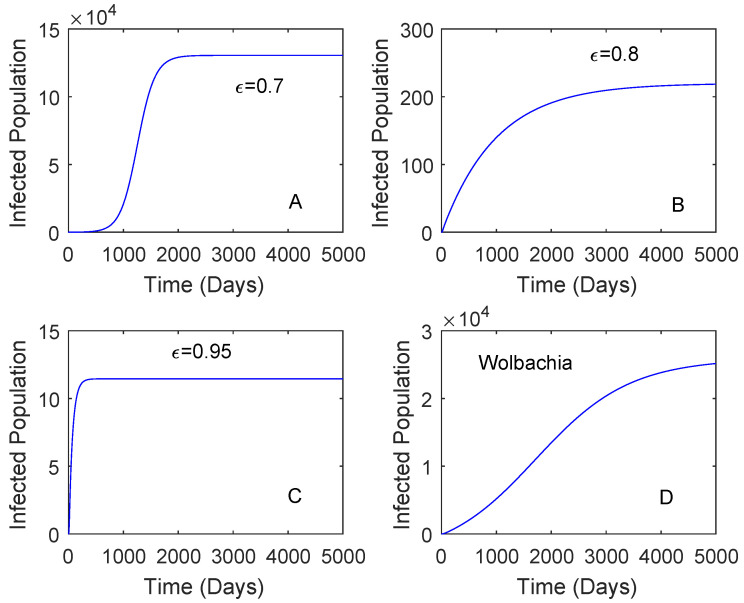
Comparing the performance of vaccination and *Wolbachia* with different vaccine efficacy. This is for the case $R_{A}=2.91$ and the vaccine rate is 0.5. The vaccine efficacy is 0.7 plot (**A**), 0.8 plot (**B**), 0.95 plot (**C**). The plot (**D**) is *Wolbachia*.

Figure 3 showed when the vaccination rate is different and the vaccine efficacy is 0.536. Although the vaccination rate is high, the performance of *Wolbachia* is still better than that of vaccine. This may be affected by a low vaccine efficacy which can lead to reinfection of vaccinated individuals. When the vaccination rate is 0.5 and the vaccine efficacy is 0.8, the reduction in the number of dengue cases by the use of vaccine is higher compared to that of *Wolbachia* as given in Figure 4. This means that the vaccine efficacy and the vaccination rate should be considered to implement a vaccination strategy. Further explanation of these parameters is given in Section 2.3.2.

#### 2.3.2. Parameter Exploration

The comparison of the performance of *Wolbachia* and vaccine is presented. We define the ‘performance index’ as follows:

(5) $$Idx_{VW}=\frac{cum_{IV}}{cum_{IW}},$$

where $cum_{IV}$ and $cum_{IW}$ are the cumulative number of infected individuals at the end of the period with vaccination strategy, and *Wolbachia* strategy, respectively. Here, we vary the vaccination rate (p) and the vaccine efficacy (ϵ). If the values of ‘performance index’ is less than unity, the cumulative number of infected individuals with the vaccination strategy is lower than that of the *Wolbachia* strategy.

**Figure 5:**
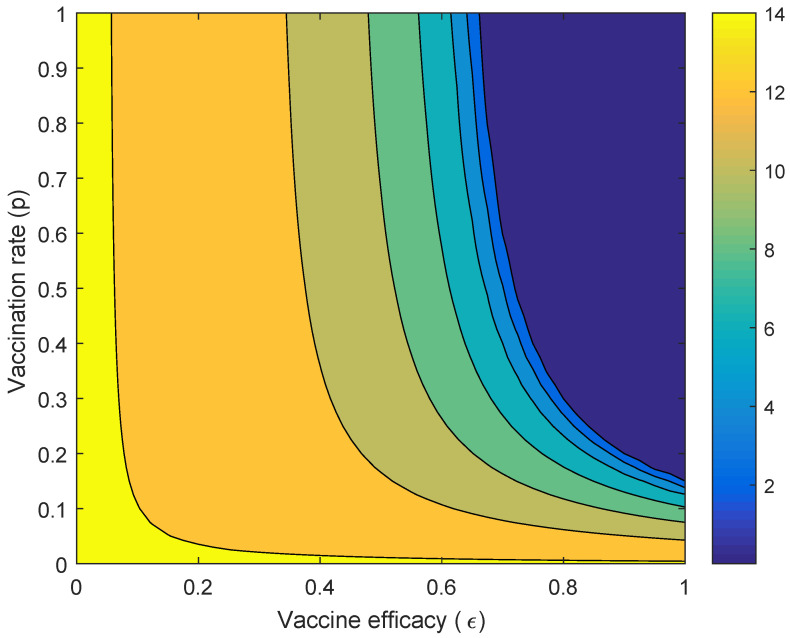
Countour plot of vaccine efficacy (ϵ) and the vaccination rate (p) against the performance index ($Idx_{VW}$).

Figure 5 presents the performance index when the vaccine efficacy and the vaccination rate are varied. It showed that when the vaccine efficacy is almost perfect but the vaccination rate is low (around 0.1), the performance of *Wolbachia* is better than the vaccine in reducing the number of dengue cases. It shows that, when the vaccine efficacy is higher (around 0.8), the vaccination rate should be around 0.5 to obtain higher reduction in the number of dengue cases compared to *Wolbachia*. When the vaccine efficacy is around 0.4, the performance of *Wolbachia* is better than that of the vaccine, although the vaccination rate is close to one.

## 3. Discussion and Conclusions

A mathematical model in the presence of vaccination and *Wolbachia* has been developed, and a global sensitivity analysis has been performed to determine the most influential parameters of the model. The performance of vaccination and *Wolbachia* in reducing the number of dengue cases has been investigated.

A global sensitivity analysis showed that the death rate of non-*Wolbachia* and *Wolbachia* carrying mosquitoes, the vaccine efficacy, the biting rates, and the transmission probability are the influential parameters on the increase number of infected individuals. The first three parameters have negative relationships, and the rest has positive relationships. This implies that, in order to reduce the number of dengue cases, we need to increase the death rate of mosquitoes and vaccine efficacy and decrease the biting rates and the transmission probability. However, a higher increase in death rate of mosquitoes leads to the extinction of *Wolbachia*-carrying mosquito population and hence non-*Wolbachia* mosquito would dominate the population and the dengue incidence cannot be reduced. A 10% reduction in *Wolbachia*-carrying mosquito death rate is sufficient to guarantee the persistence of *Wolbachia* [7,30].

Generally, the performance of *Wolbachia* in reducing dengue transmission is better than that of vaccination if the vaccine efficacy is low. Around 80% of reduction in dengue cases can be obtained with *Wolbachia* strategy only. Research showed that this may be obtained in areas with low to moderate transmission level [6,7]. The efficacy of the dengue vaccine ranges between 42% to 80% depending on the serotypes [21]. If the vaccine efficacy gets higher, the performance of the vaccine in reducing the number of dengue cases becomes effective. If the vaccine efficacy and the vaccination rate are high, the use of vaccination is better in minimising dengue transmission compared to *Wolbachia*. The results suggest that the use of vector control such as *Wolbachia* may not be necessary if the vaccine efficacy and the vaccination rate is high. In fact, the higher vaccine efficacy can be obtained when 9–45 years of age seropositive individuals were vaccinated [13,43]. Although the use of vaccine is sufficient when the vaccine efficacy and vaccination rate are high, the vector control such as using *Wolbachia* is still needed since it reduces multiple diseases such as Zika and chikungunya [44,45].

The aim of our paper is to gain general insights of the possible effectiveness of the combination of vaccine and *Wolbachia* strategies in reducing dengue transmission, and hence a single serotype dengue model is sufficient. We used a single serotype dengue model which did not take into account the effects of secondary infections and therefore it is better to extend this work by considering the multi dengue serotypes and examining the effects of vaccination and *Wolbachia* on disease transmission dynamics. Although the combination of both strategies can minimize the dengue incidence, understanding specific-serotype difference may be needed to examine the risks of the use of them particularly for vaccines, which may increase the secondary infection incidence [13,14]. Furthermore, as the mosquito population dynamics are seasonally-dependent, the effects of seasonality need to be considered since it may affect the disease transmission dynamics. We did not consider age-dependent effects on the effectiveness of the intervention in a particular vaccine, which is more effective for 9–45 years of age. The issues are the subject of future work.

## Figures and Tables

**Table 1:** Parameter descriptions, values and sources for the model. We use “Non-W” to denote non-*Wolbachia* mosquitoes and “W” to denote *Wolbachia*-carrying mosquitoes.

Symbol	Description	Value	Unit	Source
α	Maternal transmission	0.9	N/A	[30,31]
$b_{N}$	Biting rate of non-W	0.63	day${}^{-1}$	[32]
$\gamma_{H}$	Progression rate from exposed to infectious human	1/5.5	day${}^{-1}$	[33]
$\gamma_{N}$	Progression from exposed to infectious non-W	1/10	day${}^{-1}$	[34]
$\gamma_{W}$	Progression rate from exposed to infectious	1/10	day${}^{-1}$	[34]
$\mu_{N}$	Adult mosquito death rate (non-W)	1/14	day${}^{-1}$	[35]
$\mu_{NA}$	Death rate of aquatic non-W	1/14	day${}^{-1}$	[35]
$\mu_{WA}$	Aquatic death rate	1/14	day${}^{-1}$	[35]
$\rho_{N}$	Reproductive rate of non-W	1.25	day${}^{-1}$	[36]
$\rho_{W}$	Reproductive rate W	$0.95\times \rho_{N}$	day${}^{-1}$	[30]
σ	Recovery rate	1/5	day${}^{-1}$	[33]
$T_{N}$	Transmission probability	0.2614	N/A	[7]
$\tau_{N}$	Maturation rate of non-W	1/10	day${}^{-1}$	[35]
$\tau_{W}$	Maturation rate of W	1/10	day${}^{-1}$	[35]
$T_{HW}$	Transmission probability from infectious W to human	$0.5\times T_{N}$	N/A	[37]
$\mu_{W}$	Death rate of W	1.1 $\times \mu_{N}$	day${}^{-1}$	[30,38]
$b_{W}$	Biting rates of W	$0.95\times b_{N}$	day${}^{-1}$	[39]
$\mu_{H}$	Natural death rate	$\frac{1}{\left(66.38\times 365\right)}$	day${}^{-1}$	[40]
*B*	Birth rate	$\frac{1}{\left(66.38\times 365\right)}$	day${}^{-1}$	[40]
p	Vaccination rate	0.2	N/A	[41]
ϵ	Vaccine efficacy	0.538	N/A	[17,21]
ϕ	Waning immunity	0.1	N/A	[41]

## References

[1] Bhatt S., Gething P.W., Brady O.J., Messina J.P., Farlow A.W., Moyes C.L., Drake J.M., Brownstein J.S., Hoen A.G., Sankoh O. (2013). The global distribution and burden of dengue. Nature.

[2] Achee N.L., Gould F., Perkins T.A., Reiner R.C., Morrison A.C., Ritchie S.A., Gubler D.J., Teyssou R., Scott T.W. (2015). A Critical Assessment of Vector Control for Dengue Prevention. PLoS Negl. Trop. Dis..

[3] Anggriani N., Tasman H., Ndii M.Z., Supriatna A.K., Soewono E., Siregar E. (2019). The effect of reinfection with the same serotype on dengue transmission dynamics. Appl. Math. Comput..

[4] Katzelnick L.C., Gresh L., Halloran M.E., Mercado J.C., Kuan G., Gordon A., Balmaseda A., Harris E. (2017). Antibody-dependent enhancement of severe dengue disease in humans. Science.

[5] Esu E., Lenhart A., Smith L., Horstick O. (2010). Effectiveness of peridomestic space spraying with insecticide on dengue transmission; systematic review. Trop. Med. Int. Health.

[6] Ferguson N.M., Hue Kien D.T., Clapham H., Aguas R., Trung V.T., Bich Chau T.N., Popovici J., Ryan P.A., O’Neill S.L., McGraw E.A. (2015). Modeling the impact on virus transmission of *Wolbachia*-mediated blocking of dengue virus infection of *Aedes aegypti*. Sci. Transl. Med..

[7] Ndii M.Z., Hickson R.I., Allingham D., Mercer G.N. (2015). Modelling the transmission dynamics of dengue in the presence of *Wolbachia*. Math. Biosci..

[8] Ndii M.Z., Allingham D., Hickson R., Glass K. (2016). The effect of Wolbachia on dengue outbreaks when dengue is repeatedly introduced. Theor. Popul. Biol..

[9] Ndii M.Z., Allingham D., Hickson R.I., Glass K. (2016). The effect of Wolbachia on dengue dynamics in the presence of two serotypes of dengue: Symmetric and asymmetric epidemiological characteristics. Epidemiol. Infect..

[10] Ndii M.Z., Wiraningsih E.D., Anggriani N., Supriatna A.K., Falcón-Lezama J.A., Betancourt-Cravioto M., Tapia-Conyer R. (2019). Mathematical Model as a Tool for the Control of Vector-Borne Diseases: Wolbachia Example. Dengue Fever.

[11] O’Reilly K.M., Hendrickx E., Kharisma D.D., Wilastonegoro N.N., Carrington L.B., Elyazar I.R.F., Kucharski A.J., Lowe R., Flasche S., Pigott D.M. (2019). Estimating the burden of dengue and the impact of release of wMel Wolbachia-infected mosquitoes in Indonesia: A modelling study. BMC Med..

[12] Dorigatti I., McCormack C., Nedjati-Gilani G., Ferguson N.M. (2018). Using Wolbachia for Dengue Control: Insights from Modelling. Trends Parasitol..

[13] Ferguson N.M., Rodríguez-Barraquer I., Dorigatti I., Mier-y Teran-Romero L., Laydon D.J., Cummings D.A.T. (2016). Benefits and risks of the Sanofi-Pasteur dengue vaccine: Modeling optimal deployment. Science.

[14] Aguiar M., Stollenwerk N., Halstead S.B. (2016). The Impact of the Newly Licensed Dengue Vaccine in Endemic Countries. PLoS Negl. Trop. Dis..

[15] Pasteur S. (2015). Sanofi: Dengvaxia^®^, World’s First Dengue Vaccine, Approved in Mexico. https://www.sanofi.com/en/media-room/press-releases/2015/2015-12-09-16-30-00.

[16] Gailhardou S., Skipetrova A., Dayan G.H., Jezorwski J., Saville M., Van der Vliet D., Wartel T.A. (2016). Safety Overview of a Recombinant Live-Attenuated Tetravalent Dengue Vaccine: Pooled Analysis of Data from 18 Clinical Trials. PLoS Negl. Trop. Dis..

[17] Capeding M.R., Tran N.H., Hadinegoro S.R.S., Ismail H.I.H.M., Chotpitayasunondh T., Chua M.N., Luong C.Q., Rusmil K., Wirawan D.N., Nallusamy R. (2014). Clinical efficacy and safety of a novel tetravalent dengue vaccine in healthy children in Asia: A phase 3, randomised, observer-masked, placebo-controlled trial. Lancet.

[18] da Costa V.G., Marques-Silva A.C., Floriano V.G., Moreli M.L. (2014). Safety, immunogenicity and efficacy of a recombinant tetravalent dengue vaccine: A meta-analysis of randomized trials. Vaccine.

[19] Dorigatti I., Aguas R., Donnelly C.A., Guy B., Coudeville L., Jackson N., Saville M., Ferguson N.M. (2015). Modelling the immunological response to a tetravalent dengue vaccine from multiple phase-2 trials in Latin America and South East Asia. Vaccine.

[20] Sabchareon A., Wallace D., Sirivichayakul C., Limkittikul K., Chanthavanich P., Suvannadabba S., Jiwariyavej V., Dulyachai W., Pengsaa K., Wartel T.A. (2012). Protective efficacy of the recombinant, live-attenuated, CYD tetravalent dengue vaccine in Thai schoolchildren: A randomised, controlled phase 2b trial. Lancet.

[21] Villar L., Dayan G.H., Arredondo-García J.L., Rivera D.M., Cunha R., Deseda C., Reynales H., Costa M.S., Morales-Ramírez J.O., Carrasquilla G. (2015). Efficacy of a Tetravalent Dengue Vaccine in Children in Latin America. N. Engl. J. Med..

[22] Arredondo-García J., Hadinegoro S., Reynales H., Chua M., Medina D.R., Chotpitayasunondh T., Tran N., Deseda C., Wirawan D., Supelano M.C. (2018). Four-year safety follow-up of the tetravalent dengue vaccine efficacy randomized controlled trials in Asia and Latin America. Clin. Microbiol. Infect..

[23] Ndii M.Z., Anggriani N., Supriatna A.K. (2018). Application of differential transformation method for solving dengue transmission mathematical model. AIP Conf. Proc..

[24] Ndii M.Z., Supriatna A.K. (2020). Stochastic Dengue Mathematical Model in the Presence of *Wolbachia*: Exploring the Disease Extinction. Nonlinear Dyn. Syst. Theory.

[25] Supriatna A.K., Anggriani N., Husniah H. (2016). The optimal strategy of wolbachia-infected mosquitoes release program: An application of control theory in controlling dengue disease. Proceedings of the 2016 International Conference on Instrumentation, Control and Automation (ICA).

[26] Cardona-Salgado D., Campo-Duarte D.E., Sepulveda-Salcedo L.S., Vasilieva O. (2020). Wolbachia-based biocontrol for dengue reduction using dynamic optimization approach. Appl. Math. Model..

[27] Lourenço J., Recker M. (2016). Dengue serotype immune-interactions and their consequences for vaccine impact predictions. Epidemics.

[28] Zhang H., Lui R. (2020). Releasing Wolbachia-infected Aedes aegypti to prevent the spread of dengue virus: A mathematical study. Infect. Dis. Model..

[29] Hladish T.J., Pearson C.A.B., Toh K.B., Rojas D.P., Manrique-Saide P., Vazquez-Prokopec G.M., Halloran M.E., Longini I.M. (2020). Designing effective control of dengue with combined interventions. Proc. Natl. Acad. Sci. USA.

[30] Walker T., Johnson P.H., Moreira L.A., Iturbe-Ormaetxe I., Frentiu F.D., McMeniman C.J., Leong Y.S., Dong Y., Axford J., Kriesner P. (2011). The *WMel Wolbachia* strain blocks dengue and invades caged *Aedes aegypti* populations. Nature.

[31] Hoffmann A.A., Turelli M., Harshman L.G. (1990). Factors affecting the distribution of cytoplasmic incompatibility in Drosophila simulans. Genetics.

[32] Scott T.W., Amerasinghe P.H., Morrison A.C., Lorenz L.H., Clark G.G., Strickman D., Kittayapong P., Edman J.D. (2000). Longitudinal Studies of *Aedes aegypti* (Diptera: Culicidae) in Thailand and Puerto Rico: Blood Feeding Frequency. J. Med. Entomol..

[33] Gubler D.J. (1998). Dengue and dengue hemorrhagic fever. Clin. Microbiol. Rev..

[34] Chowel G., Diaz-Duenas P., Miller J.C., Velazco A.A., Hyman J.M., Fenimore P.W., Castillo-Chaves C. (2007). Estimation of the reproduction number of dengue fever from spatial epidemic data. Math. Biosci..

[35] Yang H.M., Macoris M.L.G., Galvani K.C., Andrighetti M.T.M., Wanderley D.M.V. (2009). Assessing the effects of temperature on the population of *Aedes aegypti*, the vector of dengue. Epidemiol. Infect..

[36] Ndii M.Z., Hickson R.I., Mercer G.N. (2012). Modelling the introduction of *Wolbachia* into *Aedes aegypti* to reduce dengue transmission. ANZIAM J..

[37] Bian G., Xu Y., Lu P., Xie Y., Xi Z. (2010). The Endosymbiotic Bacterium *Wolbachia* Induces Resistance to Dengue Virus in *Aedes aegypti*. PLoS Pathog..

[38] Yeap H.L., Mee P., Walker T., Weeks A.R., O’Neill S.L., Johnson P., Ritchie S.A., Richardson K.M., Doig C., Endersby N.M. (2011). Dynamics of the “Popcorn” *Wolbachia* Infection in Outbred *Aedes aegypti* Informs Prospects for Mosquito Vector Control. Genetics.

[39] Turley A.P., Moreira L.A., O’Neill S.L., McGraw E.A. (2009). *Wolbachia* Infection Reduces Blood–Feeding Success in the Dengue Fever Mosquito, *Aedes aegypti*. PLoS Negl. Trop. Dis..

[40] BPS NTT Data Nusa Tenggara Timur. http://fs.fish.govt.nz/Page.aspx?pk=7&sc=SUR.

[41] Rodrigues H.S., Monteiro M.T.T., Torres D.F. (2014). Vaccination models and optimal control strategies to dengue. Math. Biosci..

[42] Marino S., Hogue I.B., Ray C.J., Kirschner D.E. (2008). A methodology for performing global uncertainty and sensitivity analysis in systems biology. J. Theor. Biol..

[43] Flasche S., Jit M., Rodríguez-Barraquer I., Coudeville L., Recker M., Koelle K., Milne G., Hladish T.J., Perkins T.A., Cummings D.A.T. (2016). The Long-Term Safety, Public Health Impact, and Cost-Effectiveness of Routine Vaccination with a Recombinant, Live-Attenuated Dengue Vaccine (Dengvaxia): A Model Comparison Study. PLoS Med..

[44] Moreira L.A., Iturbe-Ormaetxe I., Jeffery J.A., Lu G., Pyke A.T., Hedges L.M., Rocha B.C., Hall-Mendelin S., Day A., Riegler M. (2009). *Wolbachia* Symbiont in *Aedes aegypti* limits infection with dengue, Chikungunya, and Plasmodium. Cell.

[45] Aliota M.T., Walker E.C., Uribe Yepes A., Velez I.D., Christensen B.M., Osorio J.E. (2016). The wMel Strain of Wolbachia Reduces Transmission of Chikungunya Virus in Aedes aegypti. PLoS Negl. Trop. Dis..

